# Improvement of Surface PM_2.5_ Diurnal Variation Simulations in East Africa for the MAIA Satellite Mission

**DOI:** 10.1021/acsestair.3c00008

**Published:** 2024-01-29

**Authors:** Chengzhe Li, Jun Wang, Huanxin Zhang, David J. Diner, Sina Hasheminassab, Nathan Janechek

**Affiliations:** † Department of Chemical and Biochemical Engineering, Center for Global & Regional Environmental Research, and Iowa Technology Institute, The University of Iowa, Iowa City, Iowa 52240, United States; ‡ Jet Propulsion Laboratory, California Institute of Technology, Pasadena, California 91109, United States

**Keywords:** PM_2.5_, diurnal variation, inverse modeling, UI-WRF-Chem, Addis Ababa, Ethiopia, anthropogenic emission

## Abstract

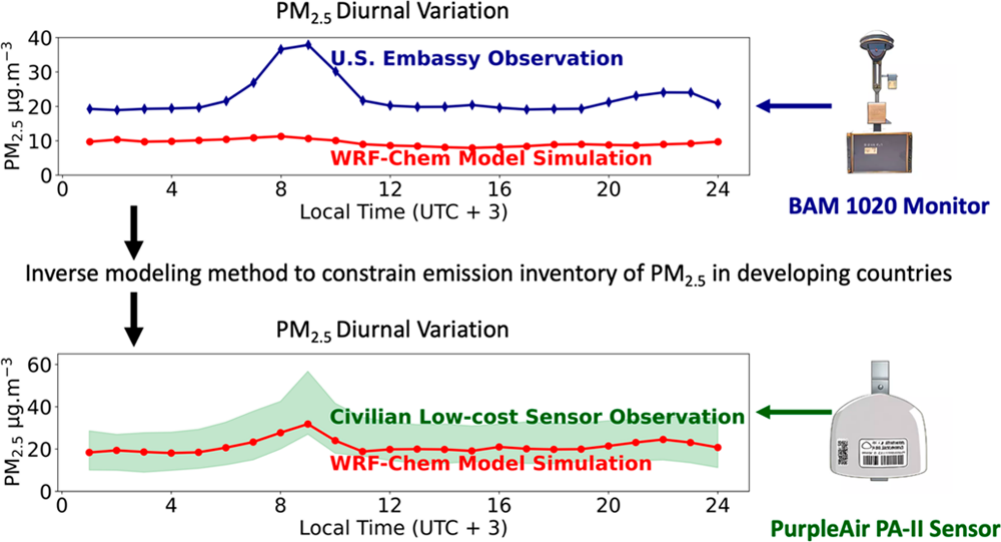

The Multi-Angle Imager for Aerosols (MAIA), supported by NASA and the Italian Space Agency, is planned for launch into space in 2025. As part of its mission goal, outputs from a chemical transport model, the Unified Inputs for Weather Research and Forecasting Model coupled with Chemistry (UI-WRF-Chem), will be used together with satellite data and surface observations for estimating surface PM_2.5_. Here, we develop a method to improve UI-WRF-Chem with surface observations at the U.S. embassy in Ethiopia, one of MAIA’s primary target areas in east Africa. The method inversely models the diurnal profile and amount of anthropogenic aerosol and trace gas emissions. Low-cost PurpleAir sensor data are used for validation after applying calibration functions obtained from the collocated data at the embassy. With the emission updates in UI-WRF-Chem, independent validation for February 2022 at several different PurpleAir sites shows an increase in the linear correlation coefficients from 0.1–0.7 to 0.6–0.9 between observations and simulations of the diurnal variation of surface PM_2.5_. Furthermore, even by using the emissions optimized for February 2021, the UI-WRF-Chem forecast for March 2022 is also improved. Annual update of monthly emissions via inverse modeling has the potential and is needed to improve MAIA’s estimate of surface PM_2.5_.

## Abstract 2

Improving air quality predictions by a combined use of PM2.5 data measured by a high-grade accurate instrument by the U.S. embassy and low-cost PurpleAir sensor data from citizens are feasible for developing countries where a surface network is not available.

## 1 Introduction

Numerous epidemiological studies have shown that respirable atmospheric particulate matter (PM) can have many effects on human health and has been associated with heart disease, stroke, lung cancer, respiratory diseases, and other adverse effects.^1−4^ Statistically, the Global Burden of Disease (GBD) study estimates that, in 2016 alone, there were more than four million premature deaths associated with exposure to ambient PM with aerodynamic diameters less than 2.5 μm (PM_2.5_).^5^ In 2016, NASA selected the Multi-Angle Imager for Aerosols (MAIA) investigation as part of the Earth Venture Instrument (EVI) program to investigate the health impacts of exposure to ambient PM.^6^ A set of 11 Primary Target Areas (PTAs) covering several highly populated cities around the world has been selected for conducting the MAIA-EVI investigation. The MAIA satellite instrument, currently planned for launch in 2025 on the Italian Space Agency’s PLATiNO-2 spacecraft, will collect targeted measurements of backscattered sunlight from which aerosol microphysical properties will be retrieved. These data will be integrated with measurements from a network of ground-based PM monitors and outputs of a chemical transport model (CTM) in a geostatistical regression model (GRM) to generate daily maps of near-surface total PM_10_, total PM_2.5_, and speciated PM_2.5_ (sulfate, nitrate, organic carbon (OC), elemental carbon (EC), and dust) mass concentrations at 1 km spatial resolution. The derived PM concentration maps will be cross-analyzed with health records to understand the association of PM mass and chemical composition with various short- and long-term health outcomes.

One of the MAIA-EVI PTAs is in Ethiopia in east Africa. This region is characterized by high cloud cover (and consequently limited availability of valid satellite measurements of aerosol optical depth data) as well as a dearth of ground-based PM observations. In other PTAs in North America, Europe, the Middle East, East Asia, and Southeast Asia there already exists a wealth of datasets and research on particulate air pollution from the past two decades.^7−13^ In Ethiopia, however, there was no observational network of surface PM_2.5_ before 2016. As of 2022, to our knowledge, the only hourly observations of surface PM_2.5_ concentrations from reference grade monitors using beta attenuation monitoring (BAM) technique, with a duration of more than one year and for which the data are publicly available, are from the U.S. Embassy Central and Jacros sites in Addis Ababa.

In the Ethiopia PTA, the sparsity of the aerosol measurements from space (due to the high cloud cover) and the lack of a surface PM_2.5_ monitoring network effectively makes the MAIA’s production of surface PM_2.5_ data rely heavily on the CTM outputs to provide fidelity as well as spatial and temporal coverage. Here, with limited surface observations in Addis Ababa, we conduct the first attempt to improve the simulation of surface PM_2.5_ by a Unified Inputs (initial and boundary conditions) for Weather Research and Forecasting model coupled with Chemistry (UI-WRF-Chem) that is customized for MAIA data production. UI-WRF-Chem is developed upon the standard version of WRF-Chem 3.8.1. WRF-Chem is an online meteorology and chemistry model that simulates meteorological fields and aerosol concentration, vertical distribution, and speciation simultaneously.^14^ WRF-Chem has been widely applied to research on the temporal and spatial variation of PM_2.5_.^15−18^

We focus on improving the diurnal variation of surface PM_2.5_ in UI-WRF-Chem in Ethiopia by updating its emissions inventory. The fidelity of any CTM simulation is limited by many factors, such as the parameterization accuracy for physical and chemical processes, the time lag of emission inventories, and the accuracy of estimation of model initial and boundary conditions.^19−21^ In the past decade, as one of the countries with a fast urbanization rate and the highest urban population growth rate in Africa, Ethiopia has experienced rapid growth in the anthropogenic PM_2.5_ emission rate.^22,23^ The primary contributors to air pollutant emissions in Ethiopia include motor vehicles, industrial sources, biomass burning, waste incineration, and dust.^24,25^ In particular, in its capital city, Addis Ababa, anthropogenic emissions account for over 95% of total aerosol and trace gas emission and contribute 85 to 93% to PM_2.5_ mass concentration (based on the model sensitivity results for the time period of study, see Figure S1 in Supporting Information). However, due to the temporal lag in the bottom-up estimates of emissions, the emissions inventory of PM_2.5_ in Ethiopia has not considered the growth of these anthropogenic sources in recent years and therefore is expected to be a large source of uncertainty for the simulation of UI-WRF-Chem in that region. To solve this issue, our study updates anthropogenic PM_2.5_ emission inventories using an inverse modeling method.

The rest of this Article is organized as follows. The detailed description of UI-WRF-Chem model and ground-based PM measurements, as well as the methods to calibrate the PurpleAir sensors and to update emission inventory by using the surface observations are described in Section 2. The results from UI-WRF-Chem with and without using the updated emissions, as well as the independent assessment of the results, are shown in Section 3.

## 2 Materials and Methods

### 2.1 UI-WRF-Chem

WRF-Chem is an online coupled model between Weather Research and Forecasting (WRF) and a chemistry package, which can be used for weather forecasting and simulating gas-phase chemistry and aerosol cloud–radiation interactions.^26,27^ For MAIA-EVI, we build upon standard WRF-Chem and we developed the UI-WRF-Chem, which has the ability to integrate chemical and meteorological fields from the same Earth system model outputs as self-consistent initial and boundary conditions. The datasets for updating both chemical and meteorological initial and boundary conditions include the Modern-Era Retrospective Analysis Research Application, Version 2 (MERRA-2) data and GEOS-Forward Processing (GEOS-FP) data. For this study, MERRA-2 0.5° × 0.625° reanalysis data are used for both meteorological and chemical boundary and initial conditions;^28−30^ this capability is developed in-house and has been used to study ozone and PM.^17,31^

To expedite the computations needed to generate timely CTM outputs for MAIA, the Regional Acid Deposition Model, Version 2 (RADM2) is applied for gas-phase chemistry in UI-WRF-Chem.^32^ Although more sophisticated modules are available, they are more computationally demanding. For aerosol simulations in UI-WRF-Chem, we adopt the Modal Aerosol Dynamics Model for Europe (MADE)^33^ and the Secondary Organic Aerosol Model (SORGAM).^34^ In the MADE/SORGAM scheme, different chemical components of aerosols, including sulfate, nitrate, ammonium, black carbon, organic matter, and secondary organic aerosol are simulated. Aerosol size distribution is represented using a modal approach that includes three modes (the Aitken, accumulation, and coarse modes). Each mode assumes a log-normal distribution.^35^ When calculating aerosol optical properties, the mass and number concentration of each aerosol species from these three modes are distributed into eight size bins, following a sectional approach.^36^ Internal mixing is assumed in each bin, and bulk properties such as the refractive index for each bin are based on a volumetric approximation.

**Figure 1:**
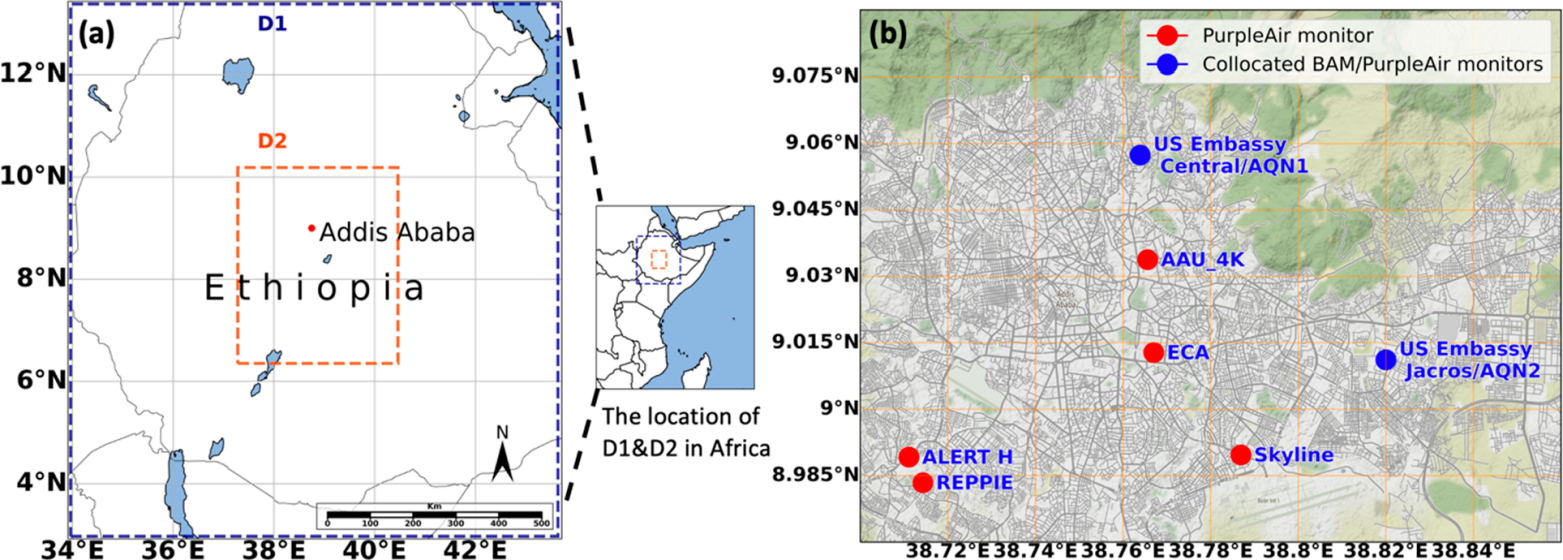
(a) Model domains. The outer domain (D1) contains major part of Ethiopia with 12 km × 12 km resolution; the inner domain (D2) includes Addis Ababa and neighboring area with 4 km × 4 km resolution. The dashed orange line shows the MAIA-EVI PTA bounding box in Ethiopia. (b) Map of ground based PM_2.5_ monitoring stations used in this study. The sites marked as red dots are PurpleAir monitors; the sites marked as blue dots are the reference BAM monitors at the U.S. Embassy and Jacros warehouse in Addis Ababa. The background street map has been obtained with permission from the open-source map dataset created by Stamen Design (http://maps.stamen.com).

For our UI-WRF-Chem simulations over Ethiopia, we set an outer domain (“D1”; Figure 1a) covering most of Ethiopia with a horizontal resolution of 12 × 12 km and a total area of 1200 km × 1200 km, and a nested inner domain (“D2”; Figure 1a). The D2 mainly covers the MAIA-EVI PTA in Ethiopia (centered at 8.2°N, 38.8°E) with a horizontal resolution of 4 km × 4 km and a total area of 360 km × 480 km. Our model divides the atmosphere into 48 vertical layers based on pressure gradients (from surface to 50 hPa) and 4 levels of soil for both domains.

We have developed the WRF Emission Preprocessing System (WEPS), an in-house utility to redistribute both global and regional anthropogenic and biomass burning emission inventories to each domain of UI-WRF-Chem.^17,31^ In this research, a 0.1° × 0.1° gridded Emissions Database for Global Atmospheric Research (EDGAR) emission inventory provided by the Task Force Hemispheric Transport of Air Pollution (HTAP or EDGAR-HTAP)^37^ is utilized for simulation in Ethiopia. The EDGAR-HTAP datasets include maps of monthly emission rates of CH_4_, CO, SO_2_, NO_*x*_, nonmethane volatile organic compound (NMVOC), NH_3_, PM_10_, PM_2.5_, Black Carbon (BC), and Organic Carbon (OC) for the years 2008 and 2010. In our WRF-Chem simulations, we exclusively utilized data from 2010 as the initial guess. For biomass burning emission inventories, we use the Fire Locating and Modeling of Burning Emissions Inventory (FLAMBE).^38^ Dust emissions follow the Goddard Global Ozone Chemistry Aerosol Radiation and Transport (GOCART) with the Air Force Weather Agency (AFWA) modifications.^39^ Prior research has substantiated the utility of these emission inventories in Africa.^40−42^ The temporal resolution of these emission inventories varies, and WEPS uses linear interpolation to assign emission rates to each hour of the simulation period. The injection height of PM_2.5_ has a large impact on model simulation and needs to be determined in WEPS. WEPS distributes anthropogenetic PM_2.5_ emission from EDGAR-HTAP inventory at the surface and fire emissions of PM_2.5_ uniformly in the model layers below the injection height. Previous studies indicate that in west Africa the injection height for smoke is on the order of 650 m,^42,43^ while other research suggests plume heights from large source complexes of >1000 m in some conditions.^44^ We adopted 800 m as the aerosol injection height in our simulation. The simulation period is from February 1 to February 28 in 2021 and February 1 to March 31 in 2022. The model spin-up period is from January 15 to January 31 in both years. During February and March, Ethiopia is in the middle of the long dry season (from October to next May), and the overall concentration of PM_2.5_ is relatively high. The model generates results for each hour. We used the model and observation data in 2021 to conduct the inverse modeling of emissions and then evaluated the robustness of the updated emissions for UI-WRF-Chem improvement in 2022.

### 2.2 Ground-Based Measurements of PM_2.5_ Concentration

Hourly surface PM_2.5_ mass concentrations, measured between 2021 and 2022 at the U.S. Embassy monitoring sites in Addis Ababa, are used as reference observations to assess the model and constrain emissions. The U.S. State Department has established two PM_2.5_ monitoring sites in Addis Ababa: one is in the north of the city, near the U.S. Embassy building; the other is in a warehouse in Jacros, east of the city. PM_2.5_ concentrations at these sites are measured by the Met One Instruments’ Model BAM-1020 Continuous Particulate Monitor, which follows the U.S. EPA Federal Equivalent Method for PM_10_ and PM_2.5_ monitoring (https://metone.com/products/bam-1020/), and the data are available on the AirNow website (https://www.airnow.gov/).

The MAIA-EVI project has installed 11 low-cost PurpleAir-II-SD (PA-II) sensors across Addis Ababa to increase the spatial coverage of surface PM_2.5_ measurements in this PTA. The PM_2.5_ measurements between February and March 2022 from seven of the PurpleAir sensors that were operational are included in this research for the independent validation of UI-WRF-Chem simulations. Figure 1b visually presents the names and locations of the BAM-1020 and PA-II sensors incorporated in this research study. PA-II sensors are strategically deployed in various locations, including urban arterial roads (“Skyline”), hospitals (“ALERT_H”), a Waste-to-Energy plant (“REPPIE”), schools (“AAU_4K”), and in collocation with BAM instruments. The selection of instrument locations is deliberate and aimed at calibrating instruments, detecting air pollutant emissions, and evaluating potential impacts on individuals who are particularly vulnerable. PurpleAir sensors have been used extensively worldwide for monitoring local PM_2.5_ concentrations and have been calibrated through various methods.^45−48^ The calibration method for the PurpleAir PM_2.5_ concentrations used in this study is described in Section 2.3.

Diurnal variations of calibrated PurpleAir PM_2.5_ data in Addis Ababa in 2022 are used to compare them with UI-WRF-Chem simulations to verify the accuracy of the model simulations. The diurnal variation of the PM_2.5_ concentration is calculated by averaging the hourly data at each hour from either model outputs or measurements. We applied several quality control methods to ensure the reliability of the measurement data from BAM and PurpleAir measurements. If there are less than 10 measurements available within a one-day period, the data of that day are rejected. If the difference in PM_2.5_ measurements between any two consecutive hours is larger than 50 μg m^–3^, the data are also rejected from the diurnal variation calculation. For PurpleAir sensors, measurements without inner temperature and relative humidity records at the same time are not accepted.

### 2.3 Correction of PurpleAir PM_2.5_ Measurements

After ground-based measurement data of the PM_2.5_ concentration from PurpleAir sensors are collected, bias corrections must be applied by using collocated reference grade sensors to derive calibration coefficients. This bias correction method takes place prior to quality assessment and data screening. Previous studies show that PurpleAir PM_2.5_ sensors generally show high correlations with reference monitors, but the accuracies are affected by environmental conditions such as temperature and relative humidity.^49^ Hygroscopic growth of particles occurs at high relative humidity and increases the reading from laser scattering particle sensors in PurpleAir. For PurpleAir measurements in Addis Ababa, two correction methods are utilized in this study. The first method applies multivariate linear regressions on collocated PurpleAir and BAM measurements at two sites in Addis Ababa (marked as U.S. Embassy Central/AQN1 and U.S. Embassy Jacros/AQN2 in Figure 1b) with the following equation:
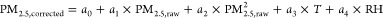
1where PM_2.5,raw_ is the hourly mean of uncalibrated PurpleAir readings in μg m^–3^ and RH is the relative humidity in percent, *T* is air temperature in °F, and *a*_0_–*a*_4_ are the calibration coefficients. *T* and RH measured by the PurpleAir sensors are used in this equation. Before calibration, the raw PurpleAir sensor data displayed a *R*^2^ of 0.70 and a RMSE of 12.46 μg/m^3^ in comparison to BAM measurements. Post-calibration, the validation dataset indicated improved performance, reflected by a *R*^2^ of 0.77 and a RMSE of 6.83 μg/m^3^ against the BAM data (see Figure S2 in Supporting Information). The second correction method adopted the work from the literature,^46^ which results from the calibration of PurpleAir sensors based on their collocations with regulatory instruments across the United States:
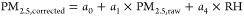
2Table 1 lists two sets of calibration coefficients for the two methods applied to PurpleAir measurements from January to March 2022.

**Table 1:** Calibration Coefficient of PurpleAir PM_2.5_ Measurements

	*a*_0_	*a*_1_	*a*_2_	*a*_3_	*a*_4_	MBE (μg m^–3^)
Method 1	15.0718	0.8613	–0.0718	0.0380	–0.3106	–3.40
Method 2	5.75	0.524			–0.0862	–16.22

### 2.4 Inverse Modeling for PM_2.5_ Emission Diurnal Profile

To improve the model simulation of diurnal variation of PM_2.5_ in Ethiopia PTA, we developed an inverse modeling technique to update the diurnal profile of anthropogenic aerosol and the trace gas emission rate related with primary PM_2.5_ in this region. Emission rates of NO, SO_2_, NH_3_, EC (also referred to as black carbon (BC)), OC, and uncharacterized PM_2.5_ are included in this research. EDGAR-HTAP emission inventory in Ethiopia PTA has four sectors: energy (power plant), industry (mining and manufacturing), transport, and residential emission. WEPS first interpolates the EDGAR-HTAP monthly emission inventory of each aerosol and trace gas from each sector linearly into daily data. Then, for the representation of the diurnal change in emission rate, we apply a diurnal profile to distribute daily data into each hour following the literature.^50,51^ The diurnal profile is normalized to ensure conservation of the emission rates.

The diurnal profile of the anthropogenic emission is updated via inverse modeling. Inverse modeling is an approach to constrain the variables in models with atmospheric observation data. For this study, we utilized the ground-based hourly measurement of PM_2.5_ concentration from the U.S. Embassy Central site in February 2021. This information allowed us to modify the diurnal profile, including the amount of the EDGAR-HTAP emission inventory in the Ethiopia PTA. It is worth noting that the Jacros site was not operational in 2021 and was not taken into consideration for inverse modeling.

We selected the 2021 data as input for the inverse modeling process and employed the 2022 observation data to validate the corresponding simulation outcomes, ensuring the avoidance of using identical observation sets concurrently for both inverse modeling and verification purposes. In this way, the stability and effectiveness of the inverse modeling can be effectively verified. For this purpose, the Jacobian matrix **K**, which gives the sensitivity of UI-WRF-Chem simulated PM_2.5_ concentration to the emission diurnal profile is defined as
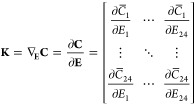
3where **C** is a vector with 24 elements representing the diurnal profile of simulated ground-level primary PM_2.5_ mass concentration at each hour (denoted with subscript number) in μg m^–3^, and **C** is calculated from the average concentration at each hour across days of the simulation; similarly, **E** represents the diurnal profile of the total emission rate of aerosols and trace gases, encompassing NO, SO_2_, NH_3_, EC, OC, and uncharacterized PM_2.5_ within the EDGAR-HTAP dataset. **E** is calculated in WEPS by applying a scaling factor to each species:
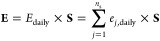
4where **S** is the vector of total scaling factor of EDGAR-HTAP at each hour and *E*_daily_ is the daily averaged emission rate in units of μg m^–2^ s^–1^ from EDGAR-HTAP, which is the sum of *e*_*j*,daily_, the emission rate of each species mentioned before, and *n*_s_ = 6 is the total number of species included in this method. Since neither PurpleAir nor BAM measures aerosol speciation, and the number of their observation sites are limited, we did not seek to use these data to directly constrain or resolve scaling factors for each sector of emissions. Rather, once hourly scaling factors in **S** for the lump sum of primary emissions from each sector are optimized from using BAM data at one embassy location only, the same set of hourly emission factors is applied to emissions from each sector. By doing so, their relative ratios of the emissions after the optimization are kept the same as those in the prior emissions of EDGAR-HTAP at each model grid box. This approach also assumes and ensures that the spatial distribution of primary emission rates from each sector is intact after the optimization. While this assumption might be incorrect, the limited observations for this study likely have insufficient information content to resolve diurnal and spatial variations of emissions among each sector, and future studies may tackle this challenge with well-designed field campaigns or observation networks for measuring aerosol speciation observations or emission for each sector in the study area.

The elements in the state vector of **S** are optimized simultaneously, with their prior values adopted from the literature.^51^ The Jacobian matrix for the Ethiopia PTA is constructed numerically from the results of several sensitivity tests of UI-WRF-Chem. In each test, a perturbation Δ*E*_*i*_ is added to emission rate *E*_*i*_ at hour *i* for each day in the simulation period by modifying the total scaling factor at that hour:
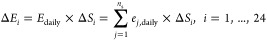
5The change in PM_2.5_ concentration, Δ**C**, can be obtained by forward simulation with and without the perturbated emission rate **E** + Δ**E**. With Δ**C**, each column of **K** is solved as
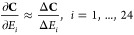
6Since each column of the Jacobian matrix is the sensitivity vector for emission rate *E*_*i*_, a maximum of 24 sensitivity simulations and one control simulation with default emission inventory are needed to construct the matrix. Preliminary tests show that if the perturbation Δ*E*_*i*_ is in range of ±*E*_*i*_ × 100%, Δ**C** exhibit a nearly linear change in response to changes in emission rate, resulting in the same value of Δ**C**/Δ*E*_*i*_. Therefore, an increment, Δ*E*_*i*_ = *E*_*i*_ × 100%, is applied to the sensitivity test. With Jacobian matrix **K** and ground-based observation data **C**_obs_, the diurnal profile of emission rate can be modified by minimizing the quadratic cost function Ψ(**E**) according to the Optimal Estimation method:^52−56^
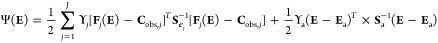
7where **F** represents the forward modeling operator, e.g., the WRF-Chem modeling, **S**_**ϵ**_ is the error covariance matrix of the observation **C**_obs_, **S**_a_ is the error covariance matrix of the a priori estimate **E**_a_, γ_*j*_ and γ_a_ are the regularization parameters, and *J* is the total number of hourly reference observations involved from the Embassy site, which is 24 × 28 for February 2021. We assume that errors are mutually independent of the diurnal factors and of the hourly observations. This assumption yields matrices **S**_**ϵ**_ and **S**_a_ with zero off-diagonal elements. Following previous studies, the diagonal elements of **S**_**ϵ**_ are set as 20% **C**_obs_, and the diagonal elements of **S**_a_ are set as 100% **E**_a_.^2,57^

Equation 7 consists of two terms on its right-hand side, each representing a specific aspect: the first term quantifies the total squared fitting error, which arises due to discrepancies between the model predictions and the observed data; the second term accounts for the penalty error, which arises when there are discrepancies between the estimates and the a priori values of emission factors. In summary, minimizing Ψ(**E**) serves the purpose of enhancing the alignment between the model and the observations while guaranteeing that the solution remains well-constrained within a practical range. The regularization parameters used in the calculation of the Ψ(**E**) function effectively balance the trade-off between the fitting error and the penalty error. In this study, we adopt an equal weighting approach for the observational constraint term and the combined a priori constraint terms within the cost function following previous studies:^54,56^
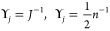
8Here *n* = 24 is the number of emission rates *E*. In essence, tackling this inverse problem equates to a purely mathematical minimization process. Iterative algorithms typically demonstrate superior numerical stability and the capability to concurrently estimate post-fitting errors. Therefore, we employ an iterative approach to determine the optimal value of *E*. A quasi-Newton approach, L-BFGS-B algorithm,^58^ is used here to minimize Ψ(*x*). The L-BFGS-B algorithm necessitates knowledge of both *x* and Ψ(*x*), along with the gradient of Ψ(*x*), which can be determined by the Jacobian matrix **K**:
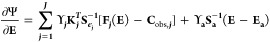
9One advantage of selecting the L-BFGS-B algorithm is that it allows implementation of bounded optimization and thus in this study prevents any component of the solution from being negative.

The iteration process halts when the reduction in Ψ(**E**) is less than 1% within a sequence of 10 consecutive iterations. After emission rate is optimized (hereafter denoted as **Ê**), emissions of each aerosol and trace gas species within each category will be increased by a part of **Ê** – **E**_a_, while maintaining the original proportions:
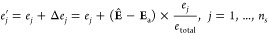
10where *e*_total_ is the sum of *e*_*j*_. Once the inversion is achieved, the inversion uncertainty can be obtained by constructing the error covariance matrix of the a posteriori state **Ŝ**:
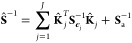
11where **K̂** is the Jacobian matrix at the solution **Ê**. After the inversion, the diagonal term of **Ŝ** is around 8.5∼19.2% of **Ê** at different hours.

This inversion can be repeated if the difference between measurement and the simulation results with an updated emission diurnal profile is still greater than the threshold (10% of **C**_obs_). In the Ethiopia PTA, our tests show that normally up to 2 iterations will be sufficient. Here, we didn’t seek to add any penalty term or high order term in eq 7 to consider either measurement uncertainty or model uncertainty, because of our limited observation as well as the high accuracy of BAM measurement (with an uncertainty of 1 μg m^–3^) and relatively high concentration of PM_2.5_ (20 μg m^–3^ in average) in our study area.

It is worth noting that eq 4 does not hold for large values of Δ**C**. Therefore, when the deficiency between simulation and measurement is larger than a certain threshold (Δ**C** > 2 × **C**_obs_ in our study), the emission rate needs to be scaled in proportion to the observed and simulated values:
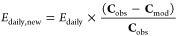
12The scaled daily emission rate *E*_daily,new_ is applied to the inverse modeling. This method also requires at least a 1 week span of each forward simulation so that the impact of changes in meteorological fields could be neglected.

## 3 Results and Discussion

After updating the diurnal (hourly) scaling factor of the EDGAR-HTAP emission inventory in February through inverse modeling for year 2021, we conducted simulations using UI-WRF-Chem for a duration of 59 days, encompassing the entire period of February and March 2022. We analyzed simulation results of these two months separately to test if the results of the diurnal profile updates in February are effective for March when no observation data were used to constrain the model.

**Figure 2:**
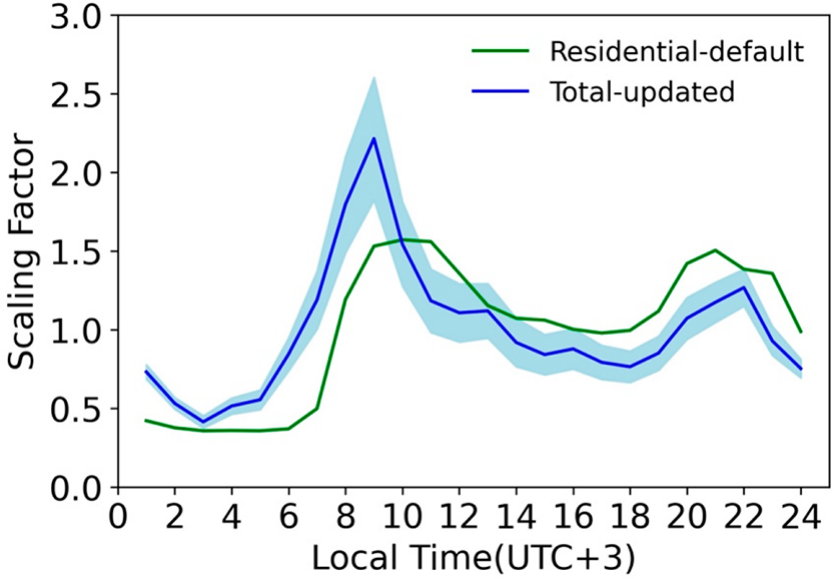
Optimized scaling factors for total emissions at each hour after applying the inverse modeling method (blue line), and default scaling factors for emission from the residential sector (green line). The light-blue shaded area is the range of 95% confidence interval of total updated scaling factor.

The revised emission diurnal scaling factor for the northwest Addis Ababa district, which encompasses the AQN1 site, is presented in Figure 2. This diurnal variation of scaling factors for the total emission at each hour is similar to the diurnal profile of residential emissions in the literature, both of which have dual peaks in the early morning and later afternoon, respectively. This similarity may also suggest the dominant contribution (>50%) from the residential sector to the total emissions of PM_2.5_, which is indeed the case in the prior emission inventory (e.g., EDGAR-HTAP). In other words, the observational constraints of surface observation may have the largest signal to constrain the diurnal variation of emissions from the residential sector. In contrast, the diurnal profiles for emissions from other sectors, while based on literature primarily for east Asia,^50,51^ could be incorrect for east Africa, which could only be a hypothesis here for future studies.

**Figure 3:**
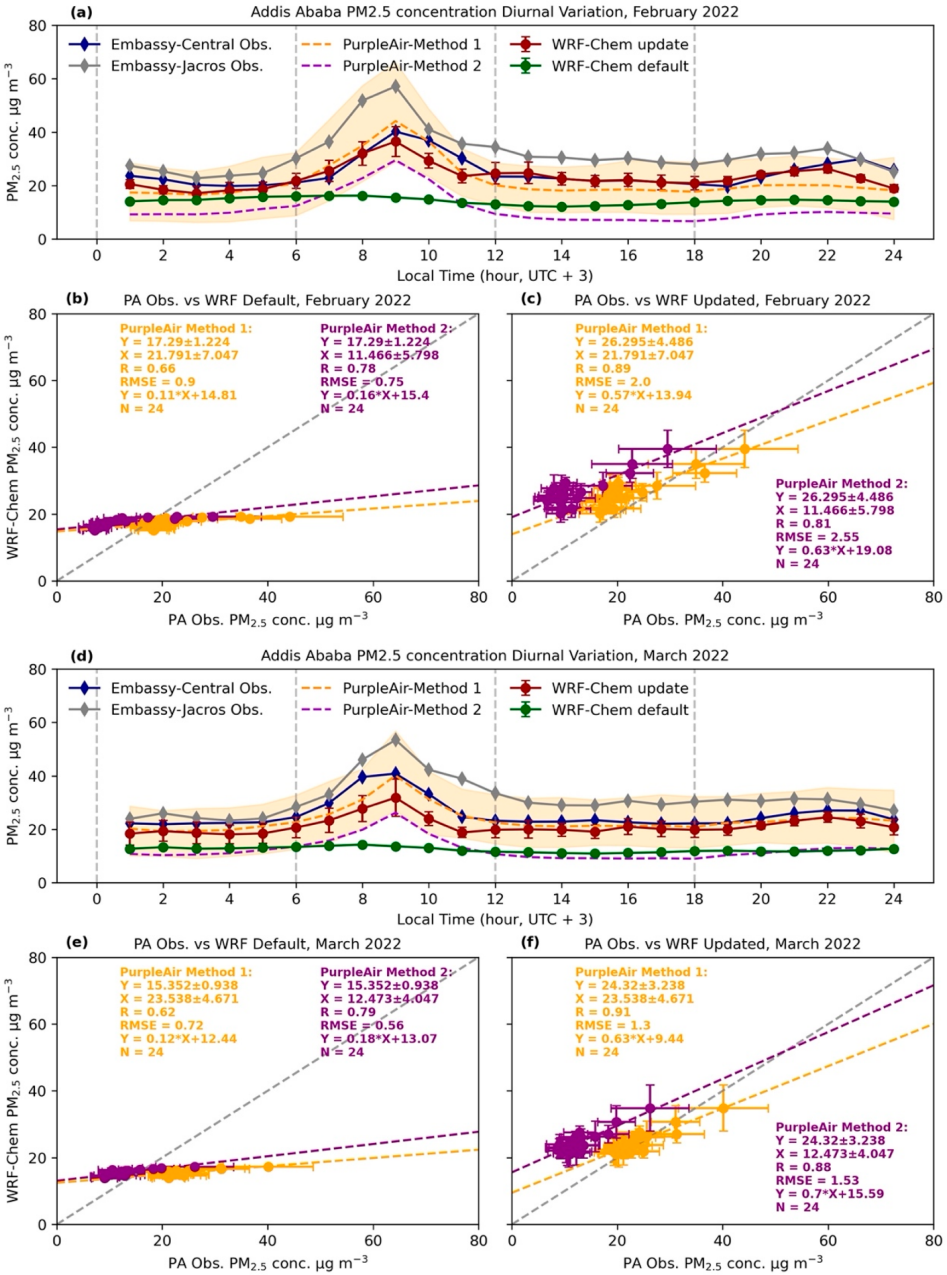
(a) Average diurnal variation of ground level PM_2.5_ concentration during February 2022 in Addis Ababa from surface measurements and UI-WRF-Chem simulations. Green dotted line represents the model simulation with default emission diurnal profile and red dotted line with updated diurnal profile. Blue and gray dotted lines represent the measurement from the BAM instrument at the U.S. Embassy Central and Jacros site. Orange dashed line and Purple dashed line represent averaged diurnal variation from seven PurpleAir measurements using the first and second correction methods related to eq 4 and eq 5, and the orange shaded area is the range of PurpleAir diurnal variation from the first correction method; (b, c) Comparison between default and updated WRF-Chem simulated diurnal variation and PurpleAir observation with calibration methods 1 (in orange) and 2 (in purple). Error bar shows the standard deviation; (d), (e), and (f) show the same results as (a), (b), and (c), but for March 2022. In the March 2022 case, the emission inventory is still updated using a U.S. Embassy Central observation in February 2021 to show the robustness of the inverse modeling method.

The comparison between the PM_2.5_ concentration diurnal variation profile from simulation and measurements in February 2022 is shown in Figure 3a–c. The model simulation data from PurpleAir and two U.S. Embassy monitors’ locations are used to calculate the mean (dots) and standard deviation (error bars) that are shown as red and green dotted lines, respectively. The diurnal variation of UI-WRF-Chem PM_2.5_ after applying the inverse modeling method (labeled as WRF-Chem update) at different hours is increased by 41∼225% (from 12.14–16.24 μg m^–3^ to 17.16–36.50 μg m^–3^) compared with a simulation using the same emission inventory but without an update (labeled as WRF-Chem default).

Diurnal variation curves of PM_2.5_ simulated by UI-WRF-Chem with an updated emission show remarkably similar patterns with the diurnal variation observed at the U.S. Embassy Central (blue dotted lines) and U.S. Embassy Jacros (gray dotted lines) and the mean of seven diurnal variation curves from the PurpleAir sensor (orange and purple dashed lines) network. Two peaks of occurrence can be observed in the same time range (East Africa time, UTC+3): one in the early morning between 8 and 9, and another in the late night between 20 and 24. The appearance of these two peaks is likely to be related to the daily activities of local citizens, corresponding to the morning traffic peak and evening residential heating and other biomass burning activities. For the simulation of the morning peak, the differences between the updated model and both the Embassy Central and PurpleAir averaged observations are less than 4 μg m^–3^, while for the simulation of the evening peak, the value from the updated model is about 5–10 μg m^–3^ higher than the average value from the PurpleAirs. The BAM observations at the U.S. Embassy Jacros site are generally higher than those of other areas, mainly because most of the industrial facilities in the city are in this area. But, this site also exhibits a similar diurnal variation pattern, supporting that the inverse modeling method works just as well.

In a stand-alone statistical analysis, the correction method calculated by local observation (Method 1) is more consistent with reference observation (comparing Mean Bias Error of Method 1 = −3.40 μg m^–3^, Mean Bias Error of Method 2 = −16.22 μg m^–3^, and Mean Bias Error of uncorrected observation = −18.82 μg m^–3^ at collocated U.S. Embassy Jacros/AQN2 site). It can also be seen from the comparison of R and RMSE of the two methods shown in Figure 3c that Method 1 is more consistent with the updated model simulation. This supports that even for a relatively mature low-cost sensor such as the PurpleAir monitor, it is also necessary to perform independent calibration on the detection results of each region. Our calibration method based on multiple linear regressions can be used in the subsequent detection of PM_2.5_ in Ethiopia PTA.

**Figure 4:**
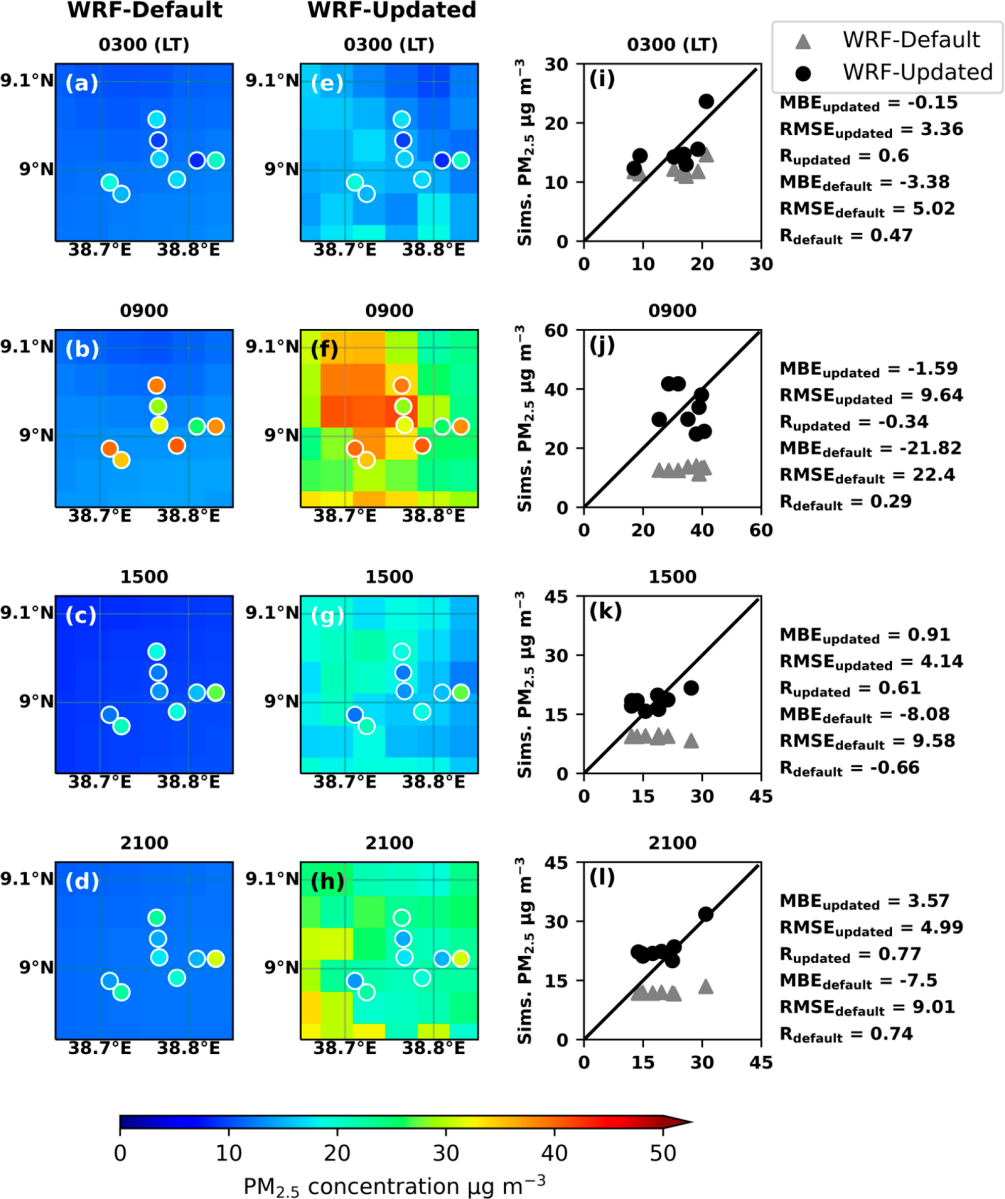
Monthly mean of February 2022 simulated (grid boxes) and observed (dots) ground-level PM_2.5_ concentration in Addis Ababa at 3 am, 9 am, 15 pm, and 21 pm. Panels a–d: UI-WRF-Chem simulation with default emission inventory; Panels e–h: UI-WRF-Chem simulation with updated emission inventory for Feb. 2021; Panel i–l: scatter plot of measured and simulated PM_2.5_ concentration at monitors’ location.

**Figure 5:**
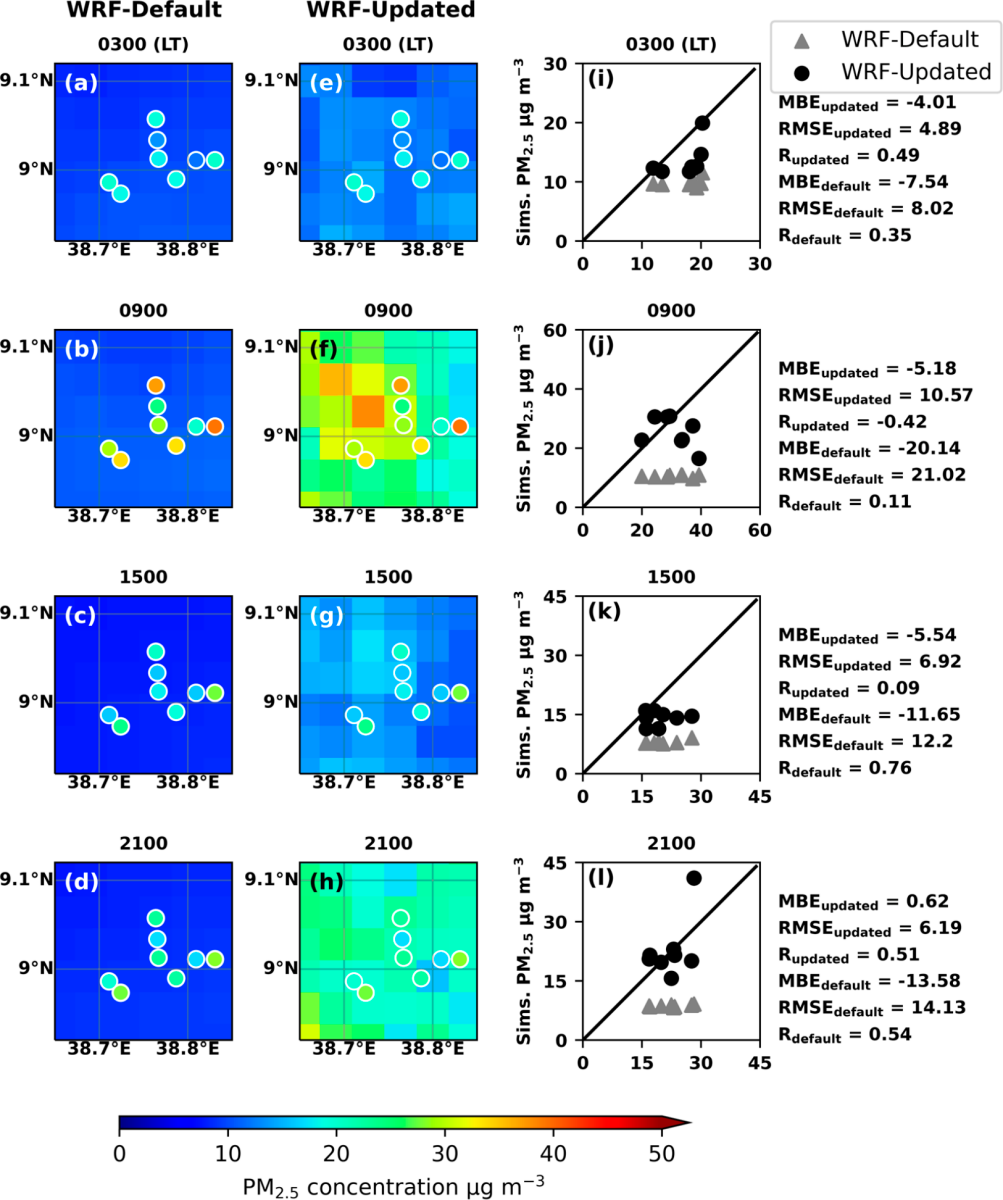
As in Figure 4, but for March 2022. Note, the emission diurnal profile used in the forecast is optimized for February 2021.

For the March 2022 simulation that uses an optimized emission for February 2021, as is shown Figure 3d–f, it still captures similar patterns of PM_2.5_ diurnal variation, as the morning peak still appears at around 8–9 and the evening peak appears at around 20–22. This consistency shows the robustness of the results in February. Figures 4 and 5 show the hourly average of simulated PM_2.5_ concentrations in Addis Ababa in February and March 2022 for four time periods, with lower PM_2.5_ concentrations at 3 and 15, and higher PM_2.5_ concentrations at 8–9 and 20. Simulations with both default and updated EDGAR-HTAP emission inventories are shown in panels a–d and e–h, while the circles in these panels represent monthly mean of ground PM_2.5_ measured at the Embassy Central, Embassy Jacros, and PurpleAir sites at each hour. UI-WRF-Chem with an updated emission inventory captures better spatial variability and higher PM_2.5_ concentrations compared to the default emission inventory. Among them, the high PM_2.5_ concentration event mainly occurs in the northwest and west of Addis Ababa. This is also the most densely populated area of the city, Addis Ketema, where the population density reaches more than 36000 people per km^2^, and the maximum PM_2.5_ value reaches around 50 μg m^–3^ (population data provided by city government of Addis Ababa: http://www.addisababacity.gov.et).

For both February and March simulations, the improved agreement between the PM_2.5_ concentration from the updated model simulation, and the observations can be further confirmed from point-to-point comparisons shown in Figures 4, 5, and S3 and S4 in Supporting Information. During the morning and evening peaks, the decreases in RMSE by applying the inverse modeling method can reach 50 to 90%, while in other hours of relatively low PM_2.5_ concentration, the decreases in RMSE are around 5–80% (panels i–l in both Figure 4 and Figure 5). Note, while the spatial distribution of the model simulated PM_2.5_ concentration is improved after emission optimization (which can be found by contrasting the first column with the second column in both Figure 4 and Figure 5), it noted that the spatial distribution is mainly determined by two factors: meteorological fields and the spatial pattern of the emission inventory. With the updated emission scaling factor, the spatial pattern of emission inventory becomes more distinct and thus increases the spatial variability of simulated PM_2.5_. EDGAR-HTAP_v2 was released in 2010, so the spatial pattern in its data could not include recent changes in anthropogenic emissions. Nevertheless, when compared to the more recent EDGAR-HTAP_v3 emission, the optimization of v2 emission here reduced its original MBE by 83% in the inner domain of this study (see Figure S5 in Supporting Information). Over the entire simulation range, our method captures the increase in emission rates with the same magnitude. To further improve the model simulation and update the spatial distribution of the emission inventory, other ancillary data are needed to further update the emission inventory, such as changes in land surface types or population density.

Simulation of anthropogenic PM_2.5_ diurnal variation is crucial to the ability of model simulation and forecast of impact of the human activity on outdoor air quality, especially in countries and areas where the PM_2.5_ concentration at the satellite overpass time is not representative of 24 h average, which is typically used for assessing daily air quality. The case study presented here presents an approach for improving model simulations based on limited surface observation of PM_2.5_. Climatic factors such as the average temperature and rainfall lead to large differences in PM_2.5_ diurnal variation between different months. Therefore, future work will be needed to further test the method and finding here for other months and in other areas. With the planned launch of MAIA, observation of PM_2.5_ speciation and more PM_2.5_ observation sites will become available, which can be used to study PM_2.5_ levels over the entire Ethiopia PTA and develop the inverse modeling method to constrain emissions from different sectors, thereby further increasing the accuracy of the simulation and forecast of PM_2.5_, including its chemical speciation, in Ethiopia.

## Data Availability

For UI-WRF-Chem modeling data, please email C.L. (chengzli@uiowa.edu) and J.W. (jun-wang-1@uiowa.edu) for details.

## Supporting Information Available

Result of the model sensitivity run with and without anthropogenic emission inventory; Correlation plot of a comparison between uncalibrated and calibrated PurpleAir observations with BAM observations; Taylor diagram of the statistical results between diurnal variation from observation and simulation with default and updated UI-WRF-Chem in February and March 2022; Emission map and correlation plot of default/updated EDGAR-HTAP v2 emission inventory and EDGAR-HTAP v3 emission inventory (DOCX)
