# Supplementary material for: Improvement of Surface PM2.5 Diurnal Variation Simulations in East Africa for the MAIA Satellite Mission
Source: ACS EST Air. 2024 Jan 29;1(4):223–33. doi: 10.1021/acsestair.3c00008 (PMC11019548; doi:10.1021/acsestair.3c00008)
Supplement: Supplementary file 1 — ea3c00008_si_001.docx [file ea3c00008_si_001.docx]

**Supporting Information for** **Improvement of surface PM_2.5_ diurnal variation simulations in East Africa for the MAIA satellite mission**

Chengzhe Li*^a^*, Jun Wang*^a^*^*^, Huanxin Zhang*^a^*, David J. Diner*^b^*, Sina Hasheminassab*^b^*, Nathan Janechek*^a^*

*^a^* Department of Chemical and Biochemical Engineering, Center for Global & Regional Environmental Research, and Iowa Technology Institute, The University of Iowa, Iowa City, 52240, Iowa, United States

*^b^* Jet Propulsion Laboratory, California Institute of Technology, Pasadena, 91125, California, United States

* Email:[jun-wang-1@uiowa.edu](mailto:jun-wang-1@uiowa.edu)

Figure S1 shows the comparison of the mean surface PM_2.5_ levels at different hours near Addis Ababa in the simulated February 2022, using UI-WRF-Chem, with and without the inclusion of the updated EDGAR HTAP_v2 anthropogenic emission inventory dataset. Panels (a)-(d) are the results without any anthropogenic emission, and panels (e)-(h) are the results with updated emission inventory. The analysis of the mean surface PM_2.5_ across the domain reveals that anthropogenic emissions constitute the primary contributor to surface PM_2.5_ levels in this region. The contribution varies between 7% and 15% at different hours.

Figure S2 shows the comparison between uncalibrated and calibrated PurpleAir PM_2.5_ and BAM PM_2.5_ observation from U.S. embassy Central and Jacros site. Using collocated BAM and PurpleAir hourly data from two U.S. embassy sites, a multivariate linear regression (MLR) was developed to correct raw PurpleAir sensor readings. The MLR model, as described by equation 1 in the paper, incorporates the raw PurpleAir data in both linear and quadratic forms. Additionally, it includes temperature and relative humidity variables, which numerous studies have identified as significant predictors for correcting PurpleAir data. The inclusion of the quadratic term is based on a study in Kanpur, India, suggesting that Plantower sensors show a nonlinear response at elevated PM_2.5_ concentrations. For this calibration, 75% of the data was considered for model training, while the remaining 25% was reserved for validation. Before calibration, the raw PurpleAir sensor data displayed an R^2^ of 0.70 and an RMSE of 12.46 µg/m³ in comparison to BAM measurements. Post-calibration, the validation dataset indicated improved performance, reflected by an R^2^ of 0.77 and an RMSE of 6.83 µg/m³ against the BAM data.

Figure S3 and S4 show Taylor diagrams^1^ that describe the statistical results between diurnal variation from observation and simulation with default and updated UI-WRF-Chem in February and March 2022, respectively. Data points in the green box represent the statistical result from comparison of simulations using the default emission inventory with observation data, and points in red box for the evaluation of simulations with the updated emission inventory. Only the simulated data from modeling grid boxes collocated with the observation site’s locations was selected in this comparison. Since the major goal of our research is to improve the simulation of diurnal variation, in these figures, the correlation and normalized standard deviation and normalized bias are calculated from diurnal variation of each model grid box relative to the mean of diurnal variation of all involved grid boxes. Here, we see that correlation, normalized standard deviation, and normalized bias increased after updating the emission inventory. The increase of correlation coefficient (from 0.12-0.72 to 0.60-0.92 in February) clearly shows that the updated model can simulate PM_2.5_ diurnal variation changes with higher accuracy, while the increase of normalized standard deviation (0.5-1.4 in February and 0.3-0.9 in March at different sites) and normalized bias suggest that the updated WRF-Chem tends to amplify the diurnal variation of PM_2.5_. In March, the correlations of diurnal variation are slightly decreased compared with those of February (from 0.6-0.92 to 0.48-0.8), but still within the acceptable range. Since no observations during March were included in the update of the diurnal scaling factor through inverse modeling, this result indicates that a monthly-based update of emission inventory would secure a high level of simulation accuracy. However, when the observational data may be discontinuous due to reasons such as instrument maintenance, the inverse modeling method can also ensure that in a longer time range, the simulation results would have a moderate level of accuracy.

Figure S5 shows the total emission rate of PM_2.5_, OC, EC, NOx and SO_2_ and NH_3_ in Ethiopia PTA in February from different emission inventories. EDGAR HTAP_v3 emission inventory is extrapolated to 2021, the year of sensitivity test following the annual trend extracted from monthly emission data from 2010 to 2018. In comparison to EDGAR HTAP_v3, the Mean Bias Error (MBE) is 82% smaller in the updated emission than in the default EDGAR HTAP_v2, underscoring the effectiveness of inverse modelling method.


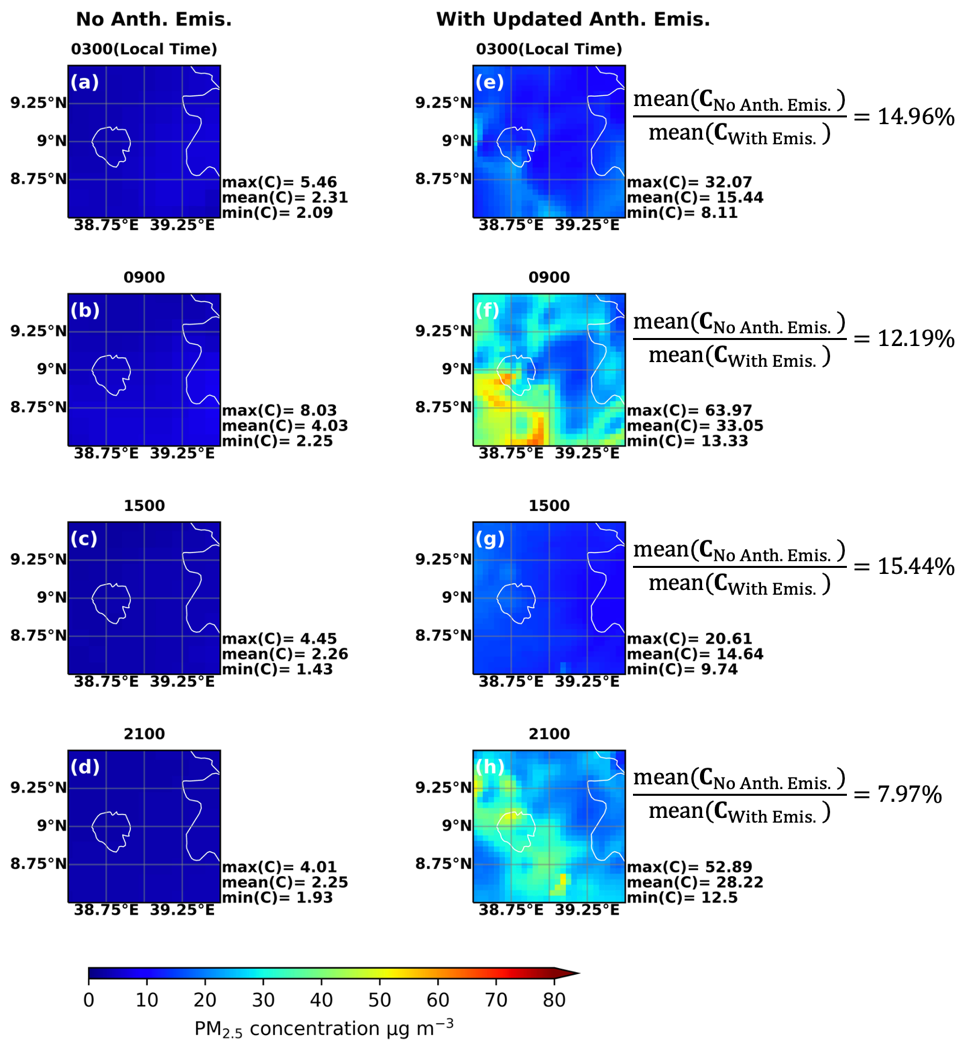


**Figure S1. Mean PM_2.5_ concentration near Addis Ababa at different hours with left panels (a-d): no anthropogenic emission added; right panels (e-h): updated anthropogenic emission.**


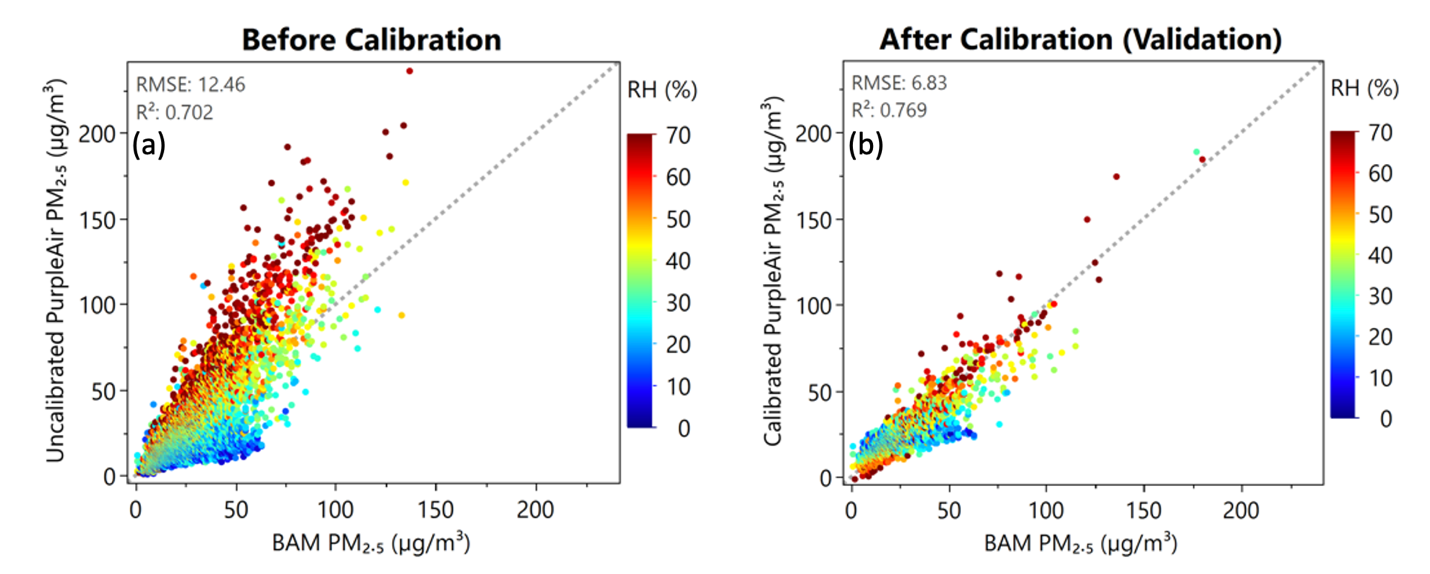


**Figure S2: Comparison between uncalibrated and calibrated PurpleAir PM_2.5_ and BAM PM_2.5_ observation from U.S. embassy Central and Jacros site.**


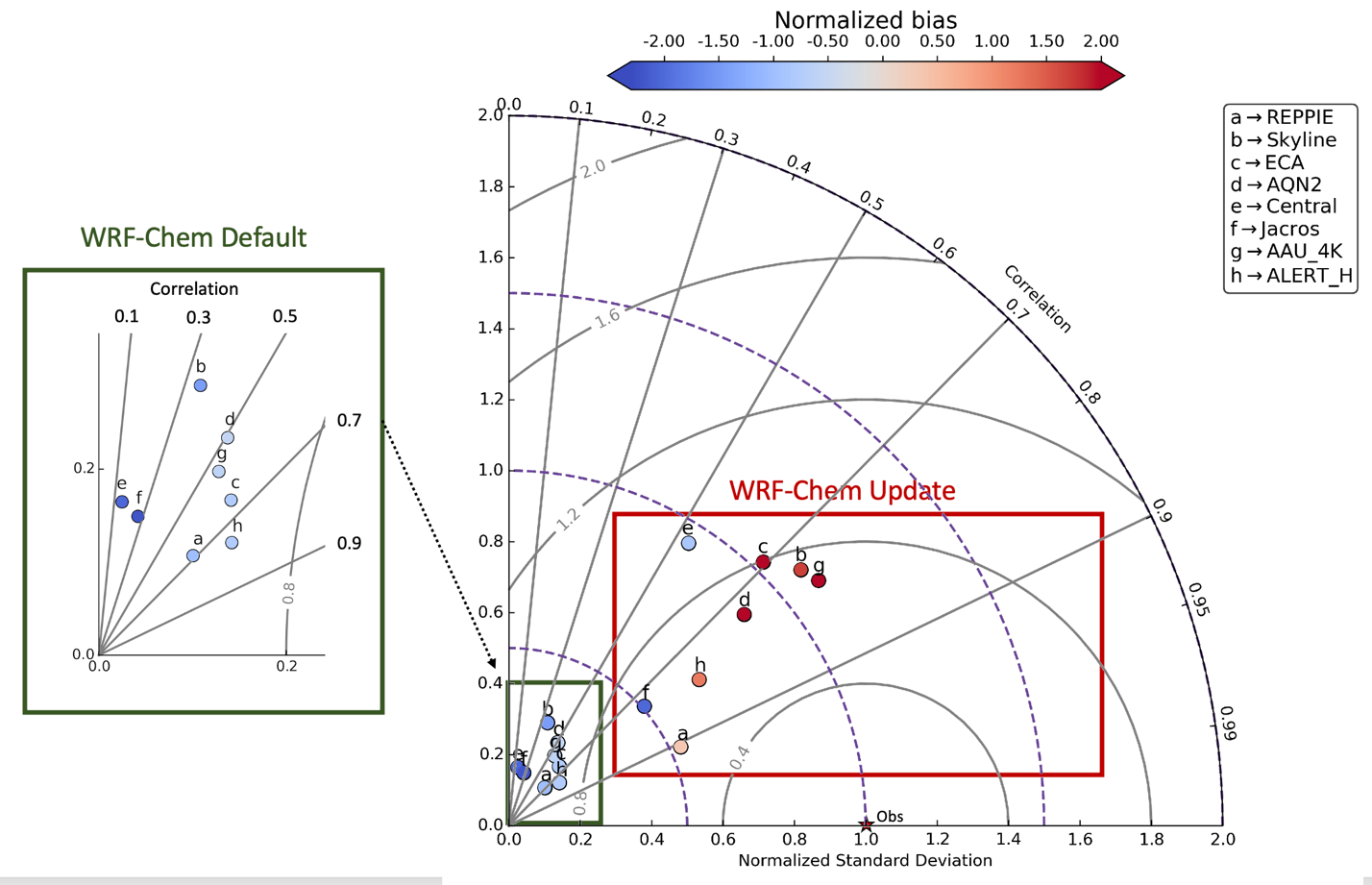


**Figure S3: Taylor Diagram of observation and simulation of PM_2.5_ concentration for February 2022. The circles represent each model comparison with ground observations. Circles in green and red box are from WRF-Chem simulation using default and updated emission inventory. ‘Obs’ represents ground observation. The colors filled in the circles denote the normalized mean bias (NMB, %).**


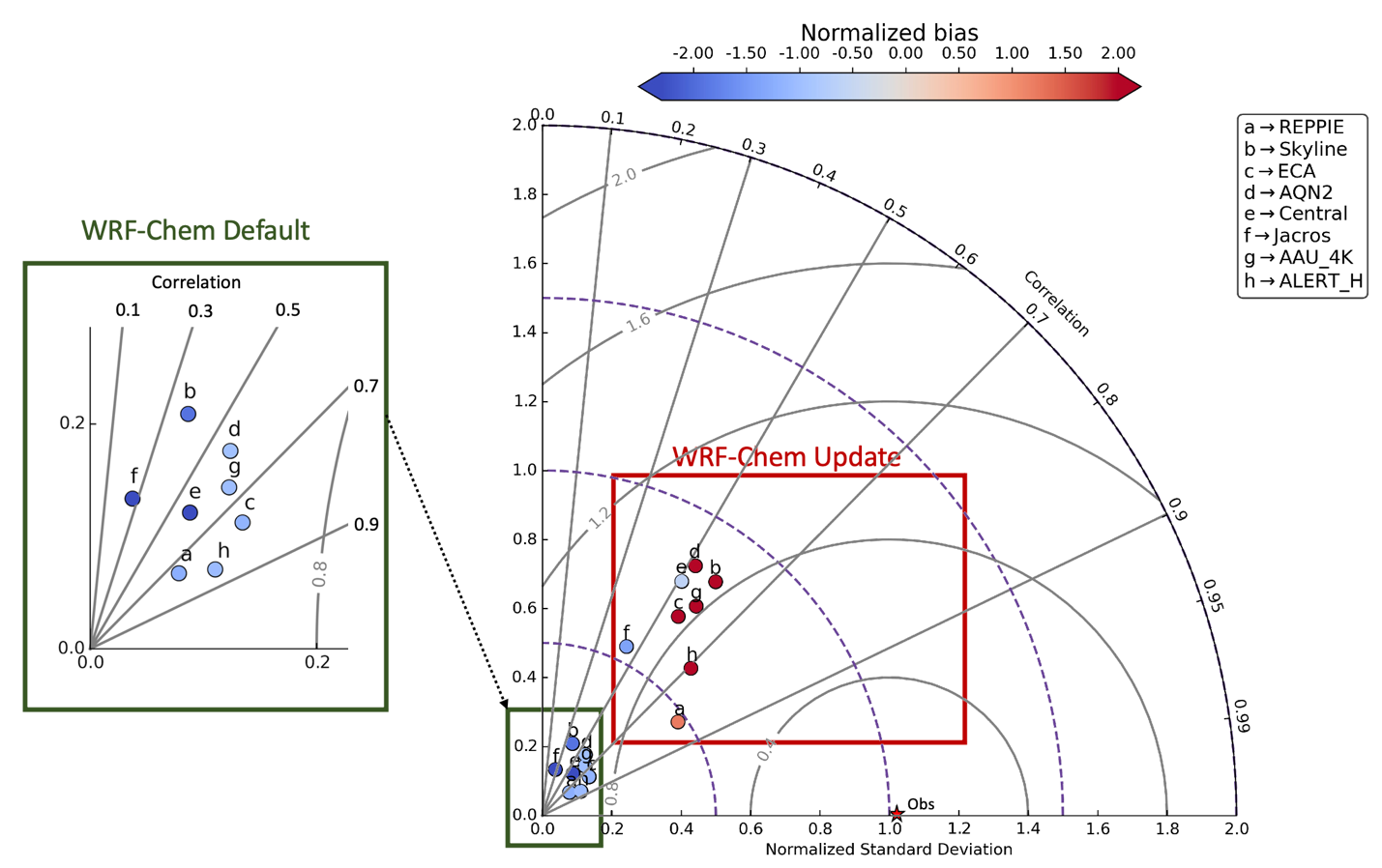


**Figure S4: As in fig S3, but for March 2022.**


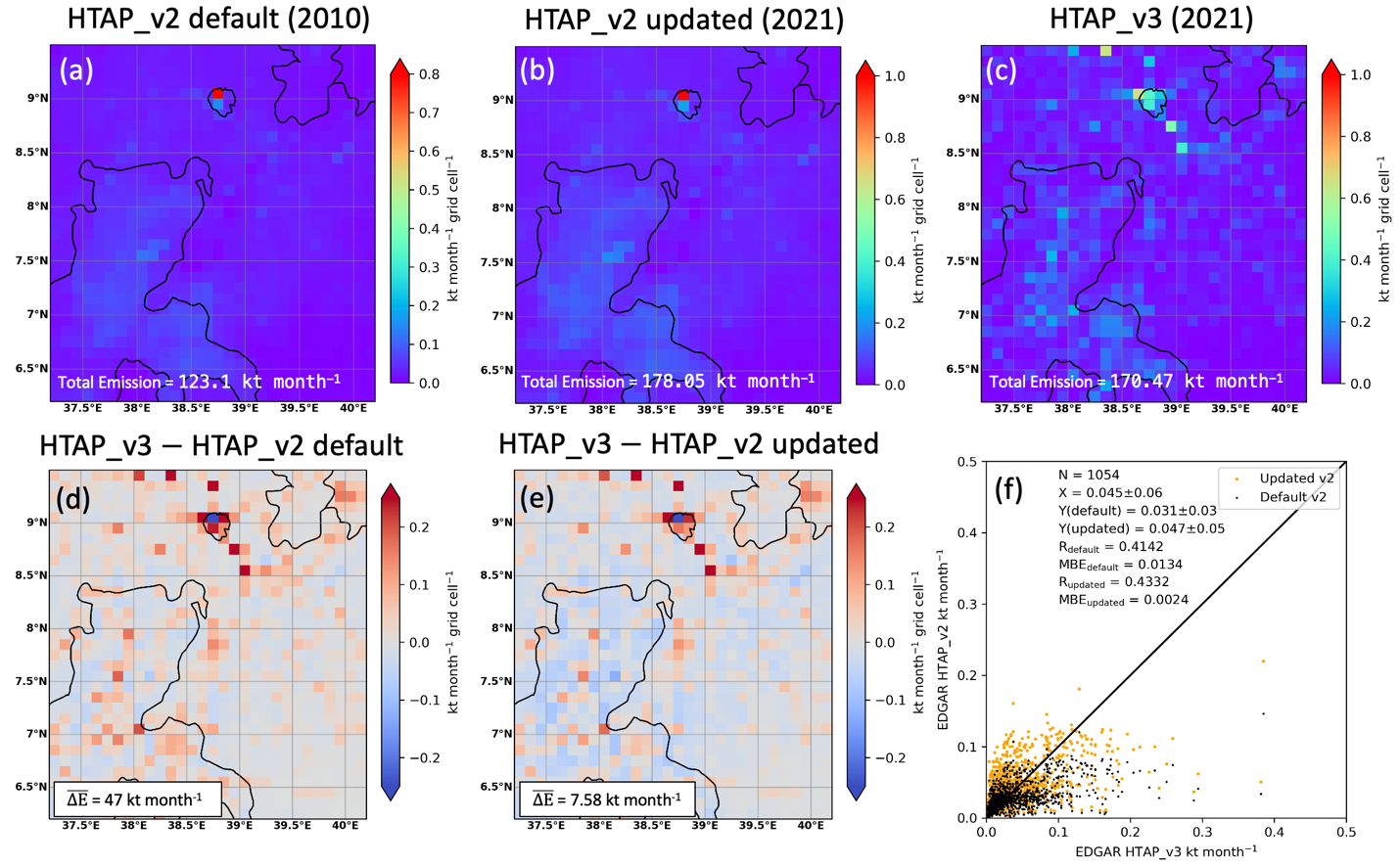


**Figure S5: Total emission rate of February 2010 from (a) EDGAR HTAP_v2 emission inventory; (b) same emission inventory but updated through inverse modelling method; (c) emission rate of February 2021; (d) and (e) difference between HTAP_v3 and default and updated HTAP_v2 inventory; and (f) comparison between updated EDGAR HTAP_v2 emission rate and EDGAR HTAP_v3.**

**Reference:**

1. Taylor, K. E., Summarizing multiple aspects of model performance in a single diagram. *J Geophys Res-Atmos* **2001,** *106* (D7), 7183-7192.
